# Comparison between hydroxychloroquine and systemic corticosteroids in IgA nephropathy: a two-year follow-up study

**DOI:** 10.1186/s12882-023-03238-7

**Published:** 2023-06-15

**Authors:** Feng-Lei Si, Chen Tang, Ji-Cheng Lv, Su-Fang Shi, Xu-Jie Zhou, Li-Jun Liu, Hong Zhang

**Affiliations:** 1 Renal Division, Peking University First Hospital, Peking University Institute of Nephrology, Beijing, China; 2 Key Laboratory of Renal Disease, Key Laboratory of Chronic Kidney Disease Prevention and Treatment (Peking University), Ministry of Health of China, Ministry of Education, Beijing, China; 3 Research Units of Diagnosis and Treatment of Immune-Mediated Kidney Diseases, Chinese Academy of Medical Sciences, Beijing, China; 4 Renal Division, Key Laboratory of Renal Disease, Peking University First Hospital, Peking University Institute of Nephrology, Ministry of Health of China, Beijing, 100034 PR China

**Keywords:** IgA nephropathy, Hydroxychloroquine, Corticosteroids, Proteinuria, Kidney function

## Abstract

**Background:**

Hydroxychloroquine (HCQ) is recommended as a treatment for IgA nephropathy (IgAN) to control proteinuria. The long-term effects of HCQ compared to systemic corticosteroid therapy remain unclear.

**Methods:**

We conducted a retrospective case‒control study at Peking University First Hospital. Thirty-nine patients with IgAN who received HCQ for at least 24 months without corticosteroids (CSs) or other immunosuppressive agents were included. Thirty-nine matched patients who received systemic CS therapy were selected using propensity score matching. Clinical data over a 24-month period were compared.

**Results:**

In the HCQ group, the level of proteinuria decreased from 1.72 [1.44, 2.35] to 0.97 [0.51, 1.37] g/d (-50.5 [-74.0, -3.4] %, P < 0.001) at 24 months. A significant decline in proteinuria was also found in the CS group, but no significant differences were found between the HCQ group and CS group in the levels of proteinuria (0.97 [0.51, 1.37] vs. 0.53 [0.25, 1.81] g/d, P = 0.707) and change rates (-50.5% [-74.0%, -3.4%] vs. -63.7% [-78.5%, -24.2%], P = 0.385) at 24 months. In addition, the decline rates of eGFR between the HCQ and CS groups were comparable (-7.9% [-16.1%, 5.8%] vs. -6.6% [-14.9%, 5.3%], P = 0.758). More adverse events were observed in the CS group.

**Conclusions:**

Long-term use of HCQ can maintain stable renal function with minimal side effects. In patients who cannot tolerate corticosteroids, HCQ might be an effective and safe supportive therapy for IgAN.

## Introduction

IgA nephropathy (IgAN) is the most common form of primary glomerulonephritis and is characterized by mesangial IgA deposition [1]. It occurs in 1 in 100 people worldwide and causes end-stage renal disease (ESRD) in Asian countries [2]. As of yet, there is no specific treatment for IgAN, which is due to the lack of knowledge about its pathogenesis [3]. To maintain the renal function of IgAN patients, supportive treatment is paramount, especially renin-angiotensin-aldosterone system inhibitors (RAASi) [4]. On the other hand, corticosteroids (CSs), another class of drugs that are widely used for IgAN, have also been shown to be effective, but the side effects cannot be ignored [5]. Therefore, an appropriate treatment is very important.

The drug hydroxychloroquine (HCQ) has been used for many years to treat malaria and was recently proposed as a possible therapy against IgAN [6]. It has been suggested that with long-term application of HCQ, a low level of proteinuria could be obtained in patients with IgAN [7]. Consequently, the 2021 Kidney Disease Improving Global Outcomes (KDIGO) Clinical Practice Guidelines recommended that HCQ could be considered in Chinese patients with IgAN, despite maximal supportive care [8]. However, when compared to CS, the effects of HCQ on IgAN remain unclear. HCQ treatment had fewer side effects after 6 months and was slightly less effective in reducing proteinuria than CS treatment in our previous study [9]. Regretfully, because of the limitation of follow-up, the long-term antiproteinuric and reno-protective efficacy comparisons between HCQ and CS therapy in patients with IgAN are unknown.

Therefore, the purpose of this study was to determine the long-term efficacy of HCQ in treating primary IgAN in comparison to systemic CS in a retrospective, propensity-matched analysis.

## Materials and methods

### Study population

This was a retrospective case‒control study performed at Peking University First Hospital. From a cohort of patients with IgAN followed in the Nephrology Department, we identified 508 patients who had received HCQ.

The inclusion criteria were as follows: (1) primary IgAN diagnosed by renal biopsy; (2) receiving HCQ treatment for at least 24 months; and (3) no CS or other immunosuppressive agents used within 3 months before or during HCQ treatment. The exclusion criteria included secondary IgAN, missing baseline or follow-up data, pregnancy or lactation, complicated with Henoch-Schönlein nephritis, chronic hepatic disease, malignant tumour, systemic lupus erythematosus or other connective tissue diseases, etc.

Similarly, we identified the patients who received the systemic CS regimen. We excluded patients treated with other immunosuppressive agents during therapy, those with crescentic IgAN, minimal changes in renal disease with IgA deposits, acute or subacute tubulointerstitial nephritis, more than 30% decline in eGFR in the previous 6 months, acute kidney injury, and malignant hypertension [9]. Furthermore, we matched patients in the HCQ and CS groups by propensity score matching based on age, sex, proteinuria level, estimated glomerular filtration rate (eGFR) and mean arterial pressure (MAP) level at the starting time of receiving HCQ or CS therapy [9]. Because the systemic steroids were given in 6- to 8-month course regimens and the enrolled patients treated with HCQ received a 24-month treatment course, we included in the analysis only those patients who had at least 24 months of follow-up from corticosteroid initiation. Furthermore, the CS therapy regimen may vary greatly depending on the patient, so we selected the most common regimens.

The study was conducted in compliance with the principles of the Declaration of Helsinki and was approved by the ethics committee at Peking University First Hospital.

### Treatment

All patients received the maximum labelled or tolerated dose of angiotensin converting enzyme inhibitors or angiotensin receptor blockers according to the KDIGO guidelines for IgAN.

In the HCQ group, the HCQ dose varied according to the baseline eGFR. The dose was 0.2 g twice daily for patients with an eGFR greater than 60 ml/min/1.73 m^2^, and the dose was 0.1 g two or three times daily for patients with an eGFR between 30 and 60 ml/min/1.73 m^2^. However, the dose was 0.1 g once daily for patients with an eGFR between 15 and 30 ml/min/1.73 m^2^ [6].

In the CS group, the “Manno regimen” (patients used prednisone or prednisolone (0.5–1 mg/kg/d; maximum, 60 mg/d) for 2 months, which was tapered to 5 mg every 2 weeks and stopped within 6 to 8 months) [10] was used.

### Study follow-up and data collection

The study follow-up period was 24 months. The following baseline characteristics were assessed: age, sex, mean arterial pressure (MAP), Oxford Classification MESTC score, 24-h total urinary protein excretion, serum creatinine level (Scr), and eGFR. MAP was calculated as diastolic blood pressure plus one-third of the pulse pressure. The eGFR was calculated by using the Chronic Kidney Disease Epidemiology Collaboration (CKD-EPI) creatinine equation [11]. The “baseline” was defined as the time at which patients started treatment with HCQ or CS. These data were collected every 1–3 months during the follow-up. All assays were performed at a laboratory in the Department of Clinical Laboratory of Peking First Hospital using standard methods.

### Study outcomes

The primary outcome was defined as the change from baseline in 24-hour proteinuria over the 24-month treatment period. The secondary outcomes involved the change in eGFR from baseline to 24 months. The slope of the decline in eGFR over two years was also calculated based on the principle of the least-squares method in a linear regression model.

The occurrence of adverse events (AEs) was also recorded. AEs were collected from the patient’s medical records and included pneumonia, gastrointestinal infection, newly diagnosed diabetes, arthralgia, palpitations, increased liver enzymes (alanine transaminase > 80 IU/mL), nausea, insomnia and skin pigmentation.

Patients receiving HCQ treatment were referred to an ophthalmologist for retinal evaluation every 3–6 months at the physician’s discretion.

### Statistical analysis

Continuous variables are expressed as either the mean ± standard deviation (SD) or median [Q25, Q75], and categorical variables are expressed as percentages. We performed chi-square tests for categorical variables and analyses of variance for continuous variables. A repeated measures analysis of variance (ANOVA) test was conducted to examine the differences between the measured variables at the different times.

We treated all missing information as missing data without imputed values. All analyses were performed using SPSS Statistics, version 22.0 (SPSS Inc., Chicago, IL, USA). P values less than 0.05 were considered statistically significant.

## Results

### Baseline characteristics

**Fig. 1:**
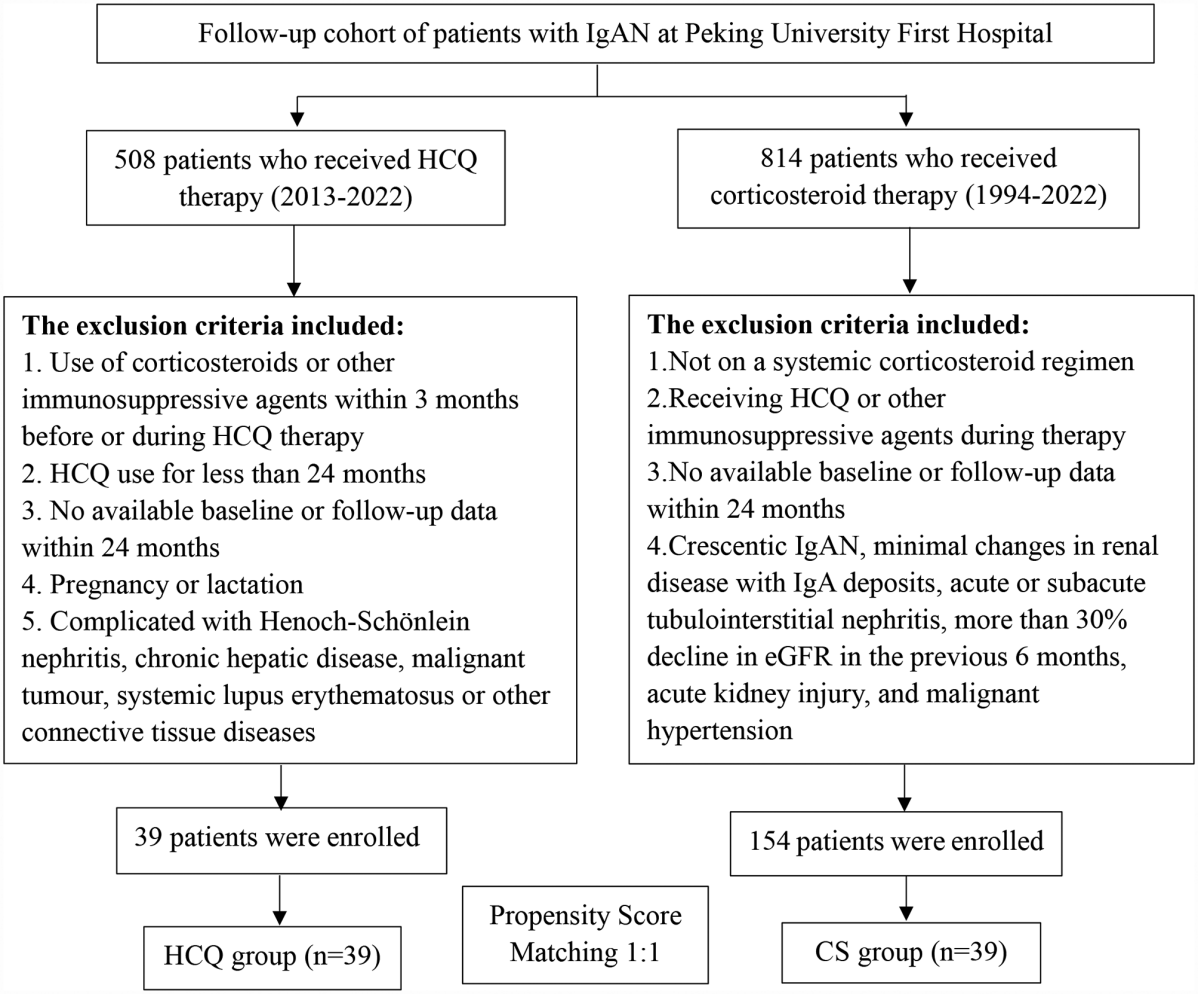
Study recruitment/inclusion flowchart

A total of 39 eligible patients with IgAN who received HCQ treatment for at least 24 months were included in this study. Thirty-nine historical controls who received systemic CS treatment were selected by propensity score matching for sex, baseline age, proteinuria, eGFR, and MAP levels (Fig. 1). All of them were treated with a full dose of the “Manno regimen”. The baseline characteristics of the two groups are shown in Table 1. The baseline proteinuria levels (1.72 [1.44, 2.35] vs. 1.86 [1.33, 2.55] g/d, P = 0.928) and eGFRs (68.54 ± 24.86 vs. 68.37 ± 21.02 ml/min/1.73 m^2^, P = 0.853) were comparable between the HCQ and CS groups.

**Table 1:** The basic characteristics of the enrolled patients

	HCQ group (n = 39)	CS group (n = 39)	P value
Gender (M/F)	22/17	23/16	0.819
Characteristics at renal biopsy			
Age (years)	33.0 ± 9.2	36.4 ± 14.7	0.666
MAP (mmHg)	93.55 ± 11.81	94.12 ± 9.60	0.912
Proteinuria (g/d)	1.44 [0.95, 2.68]	1.47 [0.70, 2.19]	0.368
eGFR level (mL/min/1.73 m^2^)	87.08 ± 29.23	78.25 ± 29.15	0.149
Serum IgA (g/L)	3.55 [2.57, 4.30]	3.25 [2.38, 3.89]	0.539
Serum C3 (g/L)	1.05 [0.91, 1.28]	0.97 [0.88, 1.16]	0.268
Interval between biopsy and HCQ/CS treatment (months)	37.4 ± 44.0	13.1 ± 27.8	< 0.001
Characteristics at baseline			
Age (years)	35.1 ± 9.1	37.5 ± 14.1	0.814
MAP (mmHg)	89.44 ± 11.30	93.11 ± 10.57	0.087
Proteinuria (g/d)	1.72 [1.44, 2.35]	1.86 [1.33, 2.55]	0.928
eGFR (mL/min/1.73 m^2^)	68.54 ± 24.86	68.37 ± 21.02	0.853
Oxford classification			
M 0/1	15/17	19/16	0.544
E 0/1	21/11	24/11	0.798
S 0/1	9/23	15/20	0.209
T 0/1/2	21/9/2	23/9/3	0.925
C 0/1/2	8/21/3	11/21/3	0.868
RAASi therapy (n, %)	39 (100.0%)	39 (100.0%)	

Histological scores of 11 patients were unavailable because they underwent renal biopsy in other clinics

### Primary outcomes

**Fig. 2:**
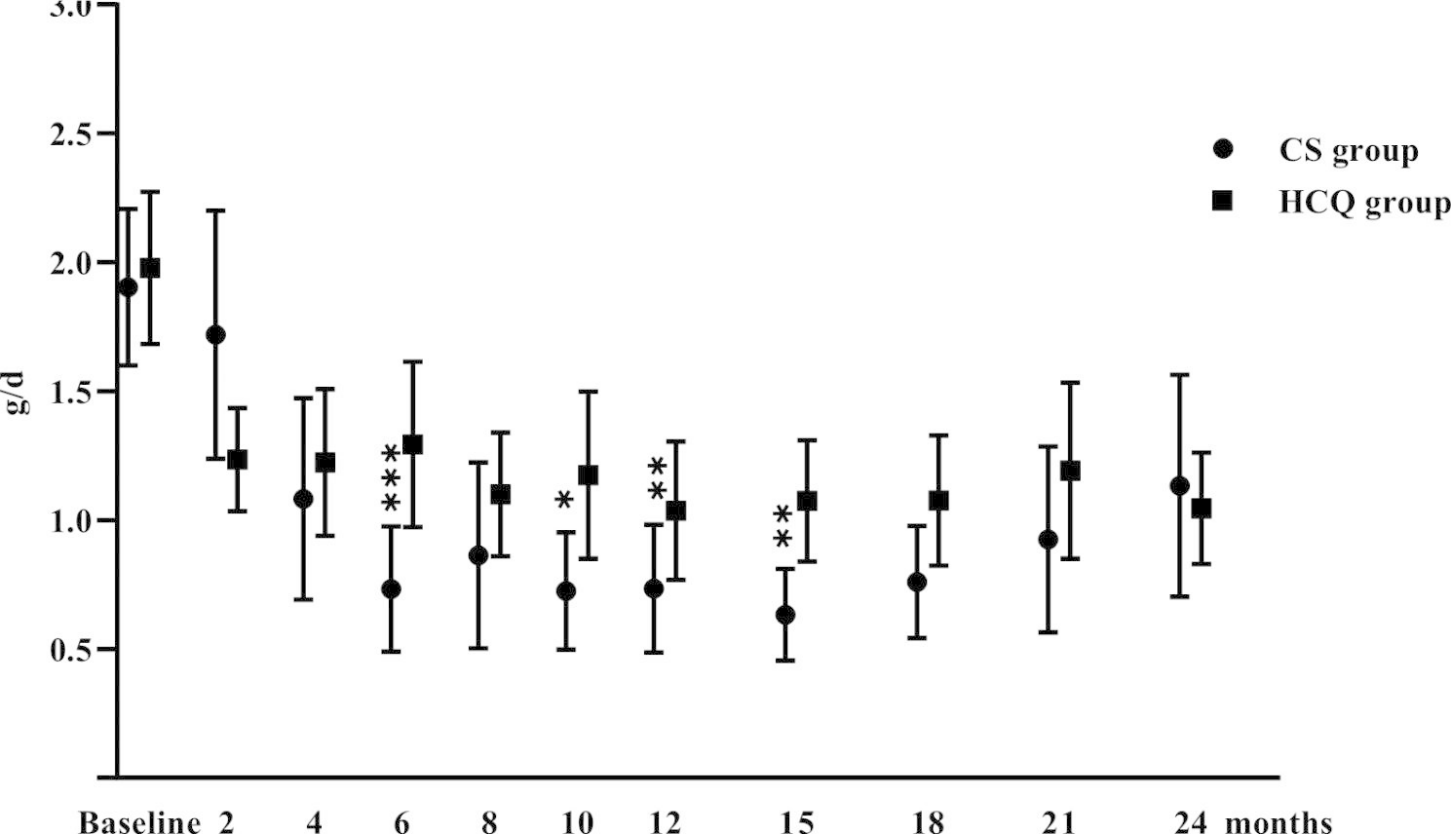
Proteinuria levels in the HCQ and systemic CS groups during the follow-up period The dots represent the mean value, and the bars represent the 95% CI. Comparisons between the two groups were made every 2–3 months *P < 0.05; **P < 0.01; ***P < 0.001

The changes in the proteinuria levels during follow-up in the two groups are shown in Table 2; Fig. 2. In the HCQ group, the level of proteinuria decreased from 1.72 [1.44, 2.35] to 0.85 [0.50, 1.41] g/d, (P < 0.001) at 12 months and to 0.97 [0.51, 1.37] g/d (P < 0.001) at 24 months. A significant decline in proteinuria was also found in the CS group, and the level of proteinuria decreased from 1.86 [1.33, 2.55] to 0.54 [0.19, 0.89] g/d (P < 0.001) at 12 months and to 0.53 [0.25, 1.81] g/d (P < 0.001) at 24 months. However, the proteinuria increased from 12 months to 24 months (0.54 [0.19, 0.89] vs. 0.53 [0.25, 1.81] g/d, P = 0.039).

**Table 2:** Primary and secondary outcomes in the HCQ and CS groups

	HCQ group(n = 39)	CS group (n = 39)	P value
**Primary outcomes**			
Proteinuria at 12 months (g/d)	0.85 [0.50, 1.41]	0.54 [0.19, 0.89]	0.099
Change in proteinuria at 12 months	-47.8% [-66.9%, -33.1%]	-73.5% [-89.5%, -57.0%]	0.005
Proteinuria at 24 months (g/d)	0.97 [0.51, 1.37]	0.53 [0.25, 1.81]	0.707
Change in proteinuria at 24 months	-50.5% [-80.6%, -3.4%]	-63.7% [-78.5%, -24.2%]	0.385
**Secondary outcomes**			
eGFR at 12 months (mL/min/1.73 m^2^)	67.54 ± 27.00	67.80 ± 23.69	0.967
Change in eGFR at 12 months	-4.4% [-15.2%, -6.3%]	-2.1% [-16.0%, 8.4%]	0.745
eGFR at 24 months (mL/min/1.73 m^2^)	66.10 ± 28.00	67.04 ± 23.80	0.877
Change in eGFR at 24 months	-7.9% [-16.1%, 5.8%]	-6.6% [-14.9%, 5.3%]	0.758
Slope of eGFR (mL/min/1.73 m^2^/year)	-1.78 ± 8.23	-1.10 ± 6.55	0.693
eGFR decrease ≥ 50% (mL/min/1.73 m^2^)	1 (3%)	2 (5%)	0.556

At 12 months, there was no significant difference between the HCQ and CS groups in the levels of proteinuria (0.85 [0.50, 1.41] vs. 0.54 [0.19, 0.89] g/d, P = 0.099), although the percentage of proteinuria reduction was smaller in the HCQ group than in the CS group (-47.8% [-66.9%, -33.1%] vs. -73.5% [-89.5%, -57.0%], P = 0.005). At 24 months, no significant differences were found between the HCQ group and CS group in the levels of proteinuria (0.97 [0.51, 1.37] vs. 0.53 [0.25, 1.81] g/d, P = 0.707) and change rates (-50.5% [-74.0%, -3.4%] vs. -63.7% [-78.5%, -24.2%], P = 0.385).

### Secondary outcomes

**Fig. 3:**
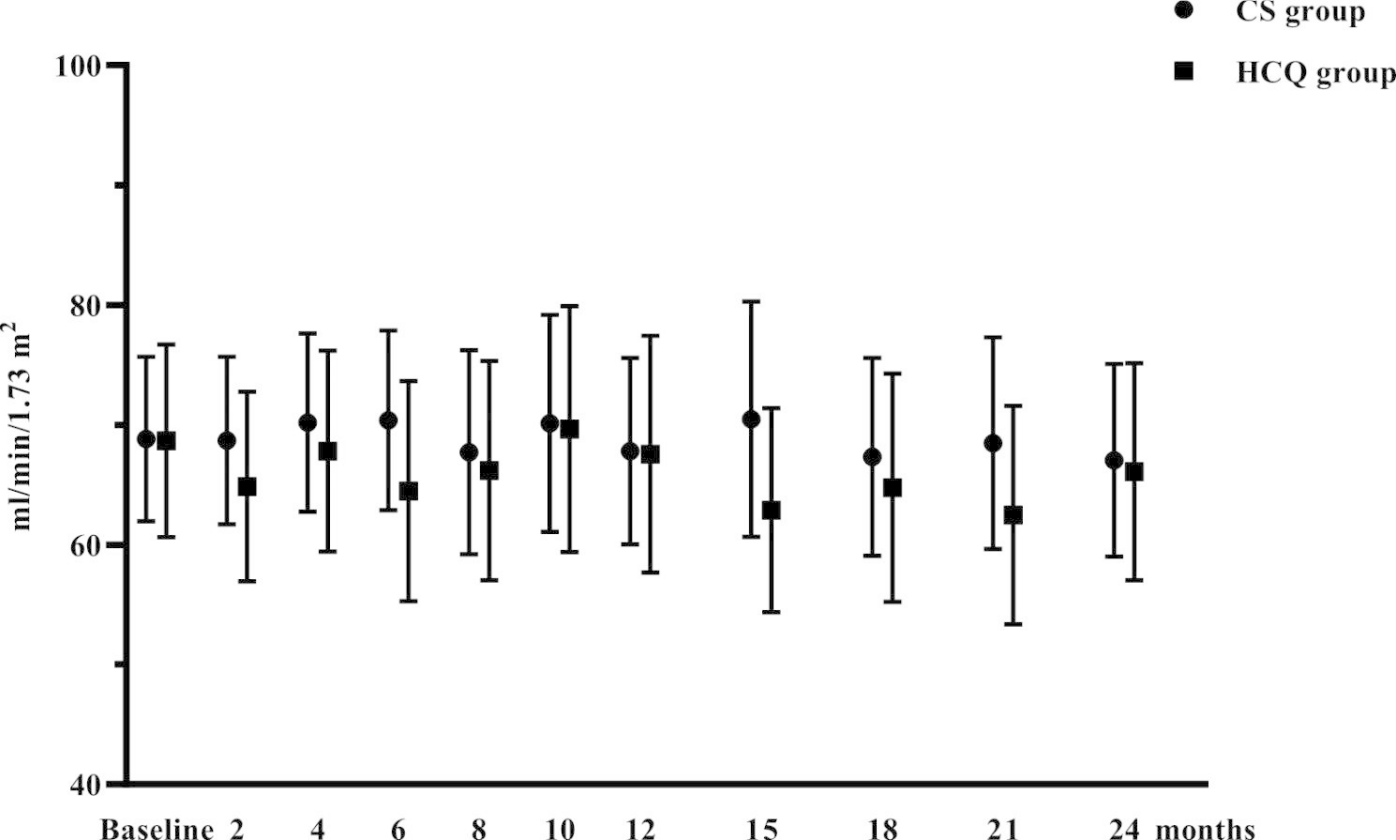
eGFR levels in the HCQ and systemic CS groups during the follow-up period The dots represent the mean value, and the bars represent the 95% CI. Comparisons between the two groups were made every 2–3 months *P < 0.05; **P < 0.01; ***P < 0.001

The changes in the eGFR levels during the follow-up are shown in Table 2; Fig. 3. During the two-year follow-up, the eGFR level in the HCQ group remained stable (68.54 ± 24.86 vs. 66.10 ± 28.00 mL/min/1.73 m^2^ at 24 months, P = 0.058). The change rates of the eGFR decline between the HCQ and CS groups did not differ significantly from baseline to 12 or 24 months (at 12 months: -4.4% [-15.2%, -6.3%] vs. -2.1% [-16.0%, 8.4%], P = 0.745; at 24 months: -7.9% [-16.1%, 5.8%] vs. -6.6% [-14.9%, 5.3%], P = 0.758, respectively). In the HCQ group, one patient (3%) experienced a more than 50% decrease in eGFR at 24 months, but 2 patients (5%) were observed in the CS group (P = 0.556). Over two years, the slope of the decline in eGFR was comparable between the HCQ and CS groups (-1.78 ± 8.23 vs. -1.10 ± 6.55 mL/min/1.73 m^2^/year, P = 0.693).

### Safety and adverse events

The AEs in both groups are listed in Table 3. During the follow-up period of two years, 5 patients (12.8%) with at least one first AE were observed in the HCQ group, and 12 patients (30.8%) were observed in the CS group (P = 0.055). In the HCQ group, AEs included nausea (2), skin pigmentation (2) and palpitations (1). No serious infections or ocular symptoms were observed. None of the patients in the HCQ group were admitted to the hospital for AEs. In the CS group, AEs mainly included infections, arthralgia, gastrointestinal discomfort and palpitations. One of them was hospitalized with pneumonia.

**Table 3:** AEs in the HCQ and systemic CS groups

	HCQ group (n = 39)	CS group (n = 39)	P value
Total AEs^a^	5 (12.8%)	12 (30.8%)	0.055
Pneumonia	0 (0)	1 (2.6%)	
Gastrointestinal infection	0 (0)	1 (2.6%)	
Newly diagnosed diabetes	0 (0)	1 (2.6%)	
Arthralgia	0 (0)	3 (7.7%)	
Palpitations	1 (2.5%)	3 (7.7%)	
Increase of liver enzymes	0 (0)	2 (5.1%)	
Nausea	2 (5.1%)	0 (0)	
Insomnia	0 (0)	1 (2.6%)	
Skin pigmentation	2 (5.1%)	0 (0)	

Multiple occurrences of the same AE in one person were only counted once

Abbreviations: AEs adverse events, ALT alanine aminotransferase

^a^ Number of patients with at least one event

## Discussion

Our study showed significant reductions in proteinuria and stabilization of renal function following 24 months of treatment with HCQ in patients with IgAN. Initially, the antiproteinuric effect of HCQ was slightly inferior to that of systemic CS treatment, but with prolonged follow-up, the effects were similar. Treatment with HCQ was well tolerated with minimal side effects. These findings suggest that HCQ might be an effective and safe supportive treatment for IgAN in patients who show intolerance to corticosteroids.

Globally, IgAN is one of the leading causes of ESRD [12]. Considering the role of immune and autoimmune activation in the pathogenesis [13], corticosteroid-based immunosuppressive treatments have been used as a potential treatment for remission in IgAN, although the high risk of adverse events also limits their use [14]. HCQ is proposed as a novel treatment for IgAN in patients who remain at high risk despite optimal supportive care [8]. Despite this, few studies have compared the effects of HCQ and CS on IgAN. In our previous retrospective study with a 6-month follow-up, we found that proteinuria in the CS group could be reduced more rapidly in the short term than in the HCQ group [9]. In the current research, patients with higher proteinuria were also included. The same trend could be observed in the first 6 months, but an upwards trend in the CS group should also be noticed in subsequent observations. There may be a “legacy” effect after the discontinuation of CS, but it eventually attenuated over time [15]. A similar pattern of early proteinuria reduction and attenuation after 3 years was observed in the STOP-IgAN trial, suggesting a relapse of histologic disease activity or progressively worsening scarring [16]. In contrast, with the continuation of HCQ, the proteinuria levels were stabilized at approximately 1 g/d in the HCQ group. Furthermore, we found that the eGFR levels remained stable in both groups with the extension of follow-up. Over two years, the slope of the decline in eGFRs was comparable between the HCQ and CS groups (-1.78 ± 8.23 vs. -1.10 ± 6.55 mL/min/1.73 m^2^/year, P = 0.693). By comparison, in a previous randomized controlled trial, the TESTING trial, patients treated with corticosteroids had an eGFR decline of -1.79 mL/min/1.73 m^2^/year [17], while in the STOP-IgAN trial, the absolute eGFR decline was − 1.4 mL/min/1.73 m^2^/year [16]. This may be related to the adjustment of medications in the real world. HCQ is a new alternative treatment for IgAN, but the time to stop its use is still uncertain. Some patients with IgAN who benefited from HCQ gradually switched to RAS inhibitors alone [7]. For patients treated with systemic CS, other immunosuppressive agents might also be used if the situation worsens. Thus, patients included in the study might respond better to CS therapy. However, in this situation, HCQ also did not appear to have a weaker effect on preserving renal function than CS therapy.

In addition, AEs should also be weighed in the treatment process. More side effects were observed in the CS group over the 24-month follow-up period, although there was no significant difference. Researchers found that a full-dose corticosteroid regimen improved proteinuria and eGFR in IgAN patients at high risk, but severe adverse effects occurred at a high rate in the TESTING trial [17]. This finding might be related to the full-dose regimen used in this study. For the HCQ group, no serious infections were reported, while significantly more skin pigmentation occurred. Currently, no significant visual or cardiac side effects have been observed, but we need to be cautious as the application time increases since toxicity is clearly correlated with usage duration [18]. Therefore, we believe HCQ could be an effective and safe supportive treatment for IgAN in patients who are corticosteroid intolerant. A few studies have also shown that HCQ is still effective in patients with large residual proteinuria who have insufficient responses to immunosuppressive therapy [19, 20].

The definite mechanism of action of HCQ in controlling IgAN is unknown. Accumulated HCQ might increase the local pH and inhibit the function of lysosomes [21]. The change in endosomal pH due to HCQ could also prevent Toll-like receptor (TLR) 9 activation [22], which is believed to influence the severity of IgAN [23]. Furthermore, HCQ might also inhibit the production of inflammatory cytokines and B-cell activating factors by inhibiting TLR pathways [24]. In addition, there is growing evidence that complement activity contributes to IgAN [25], and HCQ can also prevent complement activation [26]. However, in our study, the C3 levels were similar in both groups of patients at the time of renal biopsy, and perhaps monitoring levels during treatment will help us to find more evidence. Further elucidation of the possible mechanisms is needed.

Several limitations need to be mentioned. First, the selection bias mentioned previously was inevitable, and the sample size was relatively small. Although we matched the baseline data by propensity score matching, selection bias was inevitable. In addition, some patients did not receive HCQ immediately after diagnosis, the time from diagnosis to treatment was long in the HCQ group, and prior medication could also have affected the results to some extent. Moreover, to find a suitable match, we screened the database of patients treated with CS from 1994, whereas HCQ was only applied in the last decade; hence, more historic patients were included in the CS group. There have been advances in the management and treatment of IgAN over the past two decades, which may also affect the effectiveness of treatment. However, patients who previously had only the option of CS therapy now have more choices as well. Intervention studies with large samples are needed to verify the accuracy of the findings.

## Conclusions

In summary, the antiproteinuric effect of HCQ was slightly inferior to that of systemic CS treatment initially, but with the discontinuation of CS and the long-term application of HCQ, both have comparable effects and can maintain stable renal function. Treatment with HCQ was well tolerated with minimal side effects. These results suggest that HCQ could be an effective and safe supportive treatment for IgAN in patients who show intolerance to corticosteroids.

## Data Availability

The datasets used and/or analysed during the current study are available from the corresponding author on reasonable request.

## Abbreviations

HCQ: Hydroxychloroquine
IgAN: Immunoglobulin A nephropathy
CS: Corticosteroids
ESRD: End-stage renal disease
RAASi: Renin-angiotensin-aldosterone system inhibitor
Scr: Serum creatinine
eGFR: Estimated glomerular filtration rate
MAP: Mean arterial pressure
AEs: Adverse events
TLR: Toll-like receptor
